# Supplementary material for: Vaccinating Adolescents and Children Significantly Reduces COVID-19 Morbidity and Mortality across All Ages: A Population-Based Modeling Study Using the UK as an Example
Source: Vaccines (Basel). 2021 Oct 15;9(10):1180. doi: 10.3390/vaccines9101180 (PMC8537561; doi:10.3390/vaccines9101180)
Supplement: Supplementary file 1 [file vaccines-09-01180-s001.zip › vaccines-1396998-supplementary.pdf]

Vaccinating adolescents and children significantly reduces Covid-19 morbidity and mortality across all ages: A population-based modeling study using the UK as an example

Supplementary Appendix

Table of Contents

TRANSMISSION (SEIR) MODEL 2
DISEASE MODEL 3
TABLES 5
FIGURES 12
REFERENCES 15

List of Tables

Table S1. Population estimates for the UK 5
Table S2. Daily contacts by different age groups in the UK 5
Table S3. UK policy implementation during COVID-19 pandemic* 6
Table S4. Forecasted policy decision scenarios 6
Table S5. Proportions of hospitalized patients, patients in critical care, and deaths, stratified by age group 6
Table S6. Prevalence of variants over time (from 31st January 2021) 7
Table S7. Vaccine effectiveness against infection, hospitalizations, and deaths after the first and second dose 7
Table S8. Vaccine effectiveness against symptomatic disease and hospitalization for Alpha and Delta variants 7
Table S9. Age-dependent vaccination coverage over time to present 8
Table S10. Forecasted age-dependent vaccination coverage over time (BC scenario) 9
Table S11. Forecasted age-dependent vaccination coverage over time (BCA scenario) 10
Table S12. Forecasted age-dependent vaccination coverage over time (BCAC scenario) 11

List of Figures

Figure S1: The effective reproductive number over time since the beginning of the pandemic and assumed level for model predictions. Dots represent the observed lower and upper values of the effective reproductive number in the UK 12
Figure S2: Model trace showing the proportion of individuals who were susceptible, exposed, infectious and those who recovered from SARS-CoV-2 infection in the UK population up to December 2021. 12
Figure S3: Age dependent vaccine coverage in adults ≥18 years of age and vulnerable adolescents (BC). 13
Figure S4: Age dependent vaccine coverage in adults and all adolescents (BCA). 13
Figure S5: Age dependent vaccine coverage in adults, adolescents and children (BCAC). 14

TRANSMISSION (SEIR) MODEL

The transmission model is given by the following discrete system of nonlinear equations:

$$S_{a,t+1} = S_{a,t} - \beta\left(\frac{S_{a,t}}{N_a}\right)\sum_{j=1}^{k}\frac{M_{a,j}I_{j,t}}{\tau_I}$$

$$E_{a,t+1} = E_{a,t} + \beta\left(\frac{S_{a,t}}{N_a}\right)\sum_{j=1}^{k}\frac{M_{a,j}I_{j,t}}{\tau_I} - \frac{E_{a,t}}{\tau_E}$$

$$I_{a,t+1} = I_{a,t} + \frac{E_{a,t}}{\tau_E} - \frac{I_{a,t}}{\tau_I}$$

$$R_{a,t+1} = R_{a,t} + \frac{I_{a,t}}{\tau_I}$$

where $S_{a,t}$ is the susceptible population free from infection, $E_{a,t}$ is the exposed population but not symptomatic, $I_{a,t}$ is the symptomatic population and $R_{a,t}$ is the recovered population. The transmission rate is given by the parameter β. The length of time spent in the exposed state is given by τ_E and the length of time spent in the infectious state is given by τ_I.

The total population is given by

$$N_{pop} = \sum_{a=1}^{k} N_a = \sum_{a=1}^{k}\left(S_{a,t} + E_{a,t} + I_{a,t} + R_{a,t}\right),\ t = 0,\ 1,\ 2, \ldots,\ N_t$$

with the initial conditions given by the following:

$(R_{i,0}, E_{i,0}, I_{i,0}, R_{i,0}),\quad i = 1, 2, 3, \ldots, 18$ and $M_{i,j}$ is the population mixing patterns of the 5-year age bands, an 18 by 18 matrix.

Similar to Salje et al. 2020,[1] we adjusted our contact matrix $M_{i,j}$ to a normalized contact matrices whose maximum eigenvalues are equal to 1. We modify the contact matrix to capture the impact of the non-pharmacological interventions on the structure of the matrix. This normalization ensures that estimates of the force of infection are little impacted by the contact matrix but by the effective reproductive number.

In the UK, the virus transmission rate is unknown but the reproductive number has been estimated over time, we use the relationship[2]:

$$\beta = \frac{R_{eff}}{\tau_I \times Maximum\left(Eigenvalue\left(M_{i,j}\right)\right)}$$

where $Maximum\left(Eigenvalue\left(M_{i,j}\right)\right)$ is the maximum eigenvalue of the contact matrix $M_{i,j}$, and R_{eff} is the effective reproductive number.

DISEASE MODEL

We assume that hospitalizations (both ward and ICU) and deaths are sampled from a Poisson distribution – with the Poisson rate given as a function the number of new cases. The disease model is given by the statistical model:

$$H_{a,t} \sim Poisson\left(I_{a,t} \times \mu_{HOSP,a}\right)$$

$$LC_{a,t} \sim Poisson\left((R_{a,t} - R_{a,t-1}) \times \mu_{LCOVID,a}\right)$$

$$ICU_{a,t} \sim Poisson\left(I_{a,t} \times \mu_{IICU,a}\right) + Poisson\left(H_{a,t} \times \mu_{HICU,a}\right)$$

$$Dead_{a,t} \sim Poisson\left(I_{a,t} \times \mu_{ID,a}\right) + Poisson\left(H_{a,t} \times \mu_{HD,a}\right) + Poisson\left(ICU_{a,t} \times \mu_{ICUD,a}\right),$$

where $H_{a,t}$ is the number of new hospital admissions, $LC_{a,t}$ is the number with long COVID, $ICU_{a,t}$ is the number of new patients admitted to the ICU and $Dead_{a,t}$ is the number of new deaths from COVID disease at time t stratified by age a. The parameter $\mu_{HOSP,a}$ is the overall probability of hospitalization for COVID disease for a subject of age a, $\mu_{LCOVID,a}$ is the probability of developing long COVID, $\mu_{IICU,a}$ is the probability of ICU admission from home, $\mu_{HICU,a}$ is the probability of ICU admission for individuals currently hospitalized in wards, $\mu_{ID,a}$ is the probability of dying of COVID prior to hospitalization, $\mu_{HD,a}$ is the probability of dying for subjects hospitalized in wards and $\mu_{ICUD,a}$ is the probability of dying for individuals hospitalized in the ICU.

We use this basic model to characterise the infection since the beginning of the epidemic in the UK until to date, and then use it to predict short term dynamics and assess the implications of the upcoming UK policies on controlling the epidemic and the impact of vaccinating adolescents 12-17 and children 5-11 years old on overall disease dynamics (Figure S1 and Figure S1 show the effective reproductive number and a model trace over time, respectively).

Model parameters and data

Historical information

Disease transmission was modelled in the UK population using the age distribution of the UK 2019 mid-year populations (Table S1). Before the implementation of lockdowns, age-specific population mixing was modelled using published data (Table S2). The mixing patterns of the general population changed over the course of the pandemic in relation to changes in government policies (Tables S3 and S4). The effective reproductive number following announcement of these key changes was taken from the published data and interpolation determined the effective reproductive number for other time points.

The length of time spent by an infected individual exposed to the virus before they become infectious (latent period) was assumed to be 3 days[3]. The length of time spent by an individual in an infectious state was assumed to be 7 days[3, 4]. The probability of ward hospitalizations, ICU admission, long Covid and death are provided in Table S5. Recovered individuals were deemed to be resistant to further infection.

As shown in Table S6, only two variants have been dominant in the UK in 2021. The prevalence of individual variants was not explicitly modelled but the impact of transmission of all variants was captured using the effective reproductive number. Increased transmission due to new variants was also implicitly captured over time – for example, due to increased transmission of the delta variant, which was 7% of all sequenced samples on 18/04/2021, the effective reproductive number increases from 0.92 (observed on 12/04/2021) to 1.14 (18/04/2021), which then increases to 1.5 over time as the delta variant became dominant.

Table S7 shows vaccine effectiveness against different outcomes due to the alpha variant. A mixture of vaccines has been administered in UK with adults ≥40 years predominantly receiving the Oxford-AstraZeneca vaccine with an average efficacy against alpha variant hospitalization and deaths of 85%. Those <40 years in

the UK are vaccinated using the mRNA vaccines which have been shown to have an efficacy of 95% in preventing hospitalizations and deaths. We assumed that a single vaccine dose was associated with a 14% absolute reduction in vaccine effectiveness against symptomatic disease with delta compared to alpha, and a 10% reduction in effectiveness after 2 doses[5] (Table S8). We assume that vaccines reduce transmission by 60%[6] and long Covid by 35%[7]. Finally, vaccine coverage observed until the week 11 July 2021 is assumed to continue until the week leading up to 19 July 2021. Table S9 shows age-dependent vaccine coverage over time.

Future projections assumptions
We assume that after 19 July 2021, when restrictive measures were eased, mixing increases among the population to match pre-pandemic levels, incorporating reduced mixing among school aged children during the summer holidays with the closure of schools from 22 July 2021 and the return to school from the 6th of September 2021. The vaccine coverage by age category up to 11 July 2021 are presented in Table S9. We used the observed rate of vaccination to forecast future coverage speed. The number of people who were vaccinated between 18 July and 25 July, were assumed to have been vaccinated weekly going forward until maximum coverage for that age group

In this Supplementary Appendix, we present the input parameters and the assumptions that we used to model and predict the impact of vaccination strategies.

TABLES

Table S1. Population estimates for the UK

Age category	Population	Proportion (%)
**0-4 years**	3,857,263	5.77%
**5-9 years**	4,149,852	6.21%
**10-14 years**	3,953,866	5.92%
**15-19 years**	3,656,968	5.47%
**20-24 years**	4,153,080	6.22%
**25-29 years**	4,514,249	6.76%
**30-34 years**	4,497,132	6.73%
**35-39 years**	4,395,667	6.58%
**40-44 years**	4,019,539	6.02%
**45-49 years**	4,402,122	6.59%
**50-54 years**	4,661,015	6.98%
**55-59 years**	4,405,908	6.60%
**60-64 years**	3,755,185	5.62%
**65-69 years**	3,368,199	5.04%
**70-74 years**	3,318,867	4.97%
**75-79 years**	2,325,296	3.48%
**80-85 years**	1,715,328	2.57%
**85+ years**	1,647,271	2.47%

Source: Office for National Statistics[8]

Table S2. Daily contacts by different age groups in the UK

Age of contact	Age group of participants														
	00-04	05-09	10-14	15-19	20-24	25-29	30-34	35-39	40-44	45-49	50-54	55-59	60-64	65-69	70+
**00-04**	1.49	0.59	0.25	0.18	0.42	0.61	0.57	0.74	0.18	0.20	0.36	0.15	0.18	0.26	0.07
**05-09**	0.74	3.82	0.53	0.44	0.37	0.42	0.68	0.99	0.66	0.15	0.17	0.19	0.41	0.30	0.07
**10-14**	0.36	0.73	3.19	0.79	0.17	0.14	0.32	0.51	0.69	0.27	0.20	0.19	0.17	0.26	0.17
**15-19**	0.26	0.22	0.52	3.10	0.85	0.12	0.17	0.29	0.48	0.51	0.27	0.13	0.09	0.26	0.37
**20-24**	0.39	0.19	0.09	0.38	1.32	0.49	0.28	0.16	0.23	0.44	0.20	0.28	0.11	0.15	0.00
**25-29**	0.53	0.37	0.11	0.20	0.58	0.64	0.35	0.21	0.23	0.25	0.30	0.30	0.20	0.04	0.03
**30-34**	0.77	0.72	0.29	0.17	0.31	0.42	0.80	0.21	0.27	0.38	0.23	0.20	0.23	0.15	0.07
**35-39**	0.73	0.65	0.61	0.35	0.20	0.24	0.47	0.76	0.47	0.29	0.18	0.11	0.32	0.04	0.03
**40-44**	0.38	0.59	0.58	0.47	0.27	0.29	0.25	0.49	0.45	0.40	0.12	0.19	0.23	0.15	0.30
**45-49**	0.22	0.23	0.22	0.34	0.46	0.29	0.17	0.17	0.35	0.58	0.15	0.19	0.14	0.11	0.20
**50-54**	0.26	0.21	0.14	0.17	0.22	0.17	0.15	0.14	0.19	0.27	0.33	0.35	0.20	0.04	0.07
**55-59**	0.22	0.12	0.10	0.10	0.14	0.22	0.13	0.21	0.05	0.25	0.17	0.52	0.32	0.44	0.10
**60-64**	0.22	0.15	0.10	0.10	0.10	0.14	0.15	0.20	0.19	0.13	0.09	0.24	0.27	0.30	0.13
**65-69**	0.05	0.08	0.07	0.08	0.07	0.12	0.08	0.16	0.11	0.04	0.05	0.07	0.17	0.37	0.43
**70+**	0.09	0.09	0.10	0.08	0.19	0.10	0.05	0.13	0.24	0.29	0.23	0.22	0.11	0.22	0.70

Source: Mossong et al.[9]

Table S3. UK policy implementation during COVID-19 pandemic[*]

Event	Date	Days from start	Effective reproductive number (R_{eff})	
			Assumed[†]	Estimated range
**Pre-pandemic period**	31/01/2020	1	3.43	-
**Announcement to stop non-essential contact**	16/03/2020	40	0.70	-
**1st lockdown**	26/03/2020	55	-	-
**Phased re-opening of schools**	01/06/2020	122	-	[0.7, 0.9]
**More restrictions eased**	04/07/2020	155	-	[0.7, 0.9]
**Further restrictions eased**	14/08/2020	196	-	[0.8, 1.0]
**Impact of easing restrictions**	28/08/2020	210	1.63	[0.9, 1.1]
**New tier system begin in England**	14/10/2020	257	-	[1.3, 1.5]
**Effect of the tier system**	27/10/2020	270	0.98	[1.1, 1.3]
**2nd lockdown**	05/11/2020	279	-	[1.2, 1.5]
**2nd lockdown ends**	02/12/2020	306	-	[0.8, 1.0]
**Effect of easing 2nd lockdown**	06/12/2020	310	1.26	[0.8, 1.0]
**More areas in England enter in Tier 4**	26/12/2020	330	-	[1.1, 1.3]
**3rd lockdown**	06/01/2021	341	-	[1.1, 1.4]
**Effect of locking down**	08/01/2021	343	0.80	[1.0, 1.4]
**Effect of locking down**	21/01/2021	356	0.74	[0.8, 1.0]
**Moving out of 3rd lockdown: Step 1**	08/03/2021	402	0.78	[0.6, 0.8]
**Moving out of 3rd lockdown: Step 2**	12/04/2021	437	0.92	[0.7, 1.0]
**Delta variant in circulation: about 7%**	18/04/2021	443	1.14	[0.7, 1.1]
**Delta variant in circulation: about 42%**	08/05/2021	463	1.27	[0.8, 1.1]
**Moving out of 3rd lockdown: Step 3**	17/05/2021	472	-	[0.9, 1.1]
**Delta variant in circulation: about 62%**	18/05/2021	473	1.50	[1.0, 1.1][‡]
**Delta variant in circulation: about 95%**	07/06/2021	493	1.50	[1.1, 1.4][‡]
**Delta variant in circulation: about 100%**	05/07/2021	521	1.50	[1.2, 1.5][‡]

Source: Department of Health and Social Care[10]

[*]Note that the date of policy announcement does not imply immediate impact on transmission– this is particularly applicable to announcements of imposed restrictions. [†]The initial value used in the interpolation. [‡]England data only available

Table S4. Forecasted policy decision scenarios

Event	Days	Date	Assumed effective reproductive number
**Freedom day**	535	19/07/2021	1.4 [1.1, 1.4][‡]
**Schools close**	538	15/07/2021	1.4 [1.1, 1.4][‡]
**Schools open**	584	06/09/2021	1.4

Source: Department of Health and Social Care[10]. [‡]England data only available

Table S5. Proportions of hospitalized patients, patients in critical care, and deaths, stratified by age group

Age group	Proportion of symptomatic cases requiring hospitalization (%)	Proportion of symptomatic cases who end up dying (%)	Proportion of hospitalized cases requiring critical care (%)	Proportion of critical care patients who end up dying (%)	Proportion with long COVID (%)
**0-9 years**	0.1	0.0001	5.0	40.0	3.1*
**10-19 years**	0.3	0.0003	5.0	40.0	3.1*
**20-29 years**	1.2	0.03	5.0	50.0	3.1
**30-39 years**	3.2	0.08	5.0	50.0	4.7
**40-49 years**	4.9	0.15	6.3	48.6	4.7
**50-59 years**	10.2	0.60	12.2	48.2	3.8
**60-69 years**	16.6	2.2	27.4	48.4	3.8
**70-79 years**	24.3	5.1	43.2	48.6	1.5
**80+ years**	27.3	9.3	70.9	48.0	1.5

Source: Ferguson et al.[11]
Mortality in children taken from Smith et al. 2021[12]
Prevalence of long COVID taken from the Opinions and Lifestyle Survey[13]
**We assumed the same frequency of long COVID in children and adolescents*

Table S6. Prevalence of variants over time (from 31st January 2021)

Week	Alpha	Beta	Delta
**31/01/2021**	95.0%	0.1%	0.0%
**07/02/2021**	96.1%	0.2%	0.0%
**14/02/2021**	97.3%	0.1%	0.0%
**21/02/2021**	97.3%	0.2%	0.0%
**28/02/2021**	96.9%	0.3%	0.0%
**07/03/2021**	97.3%	0.3%	0.0%
**14/03/2021**	97.6%	0.5%	0.0%
**21/03/2021**	98.3%	0.4%	0.0%
**28/03/2021**	97.8%	0.6%	0.2%
**04/04/2021**	95.4%	1.1%	1.1%
**11/04/2021**	92.8%	0.8%	3.0%
**18/04/2021**	88.5%	0.9%	6.9%
**25/04/2021**	83.8%	0.9%	12.1%
**02/05/2021**	71.4%	0.5%	25.5%
**09/05/2021**	55.4%	0.4%	41.9%
**16/05/2021**	36.2%	0.1%	62.2%
**23/05/2021**	19.5%	0.1%	79.6%
**30/05/2021**	9.6%	0.1%	90.0%
**06/06/2021**	5.2%	0.1%	94.5%
**13/06/2021**	3.0%	0.0%	96.7%

Source: Wolfel et al.[14]

Table S7. Vaccine effectiveness against infection, hospitalizations, and deaths after the first and second dose

Endpoint	Vaccine effectiveness Pfizer-BioNTech		Vaccine effectiveness Oxford-AstraZeneca		Overall*
	One week after the first dose	One week after the second dose	One week after the first dose	One week after the second dose	
**Symptomatic disease**	55-70%	85-95%	55-70%	70-85%	72.5%
**Hospitalization**	75-85%	90-99%	75-85%	80-99%	85.8%
**Mortality**	70-85%	95-99%	75-85%	75-99%	85.0%
**Infection**	55-70%	70-90%	55-70%	65-90%	70.5%
**Transmission†**	45-50%	No data	35-50%	No data	No data

Source: Public Health England[15]
**Assuming 60% share of the AstraZeneca and 40% of the mRNA vaccines in the UK. †Secondary cases*

Table S8. Vaccine effectiveness against symptomatic disease and hospitalization for Alpha and Delta variants

Endpoint	Alpha		Delta	
	One week after the first dose	One week after the second dose	One week after the first dose	One week after the second dose
**Symptomatic disease**	49% (46 - 52)	89% (87 - 90)	35% (32 - 38)	79% (78 - 80)
**Hospitalization**	78% (64 - 87)	93% (80 - 97)	80% (69 - 88)	96% (91 - 98)

Source: Public Health England[15]

Table S9. Age-dependent vaccination coverage over time to present

Date	0-9y	10-19y	20-29y	30-39y	40-49y	50-59y	60-69y	70-79y	80+y
**27/12/2020**	0.00	0.00	0.00	0.00	0.00	0.00	0.00	0.00	0.18
**03/01/2021**	0.00	0.00	0.00	0.00	0.00	0.00	0.00	0.01	0.23
**10/01/2021**	0.00	0.00	0.00	0.00	0.00	0.00	0.00	0.01	0.37
**17/01/2021**	0.00	0.00	0.00	0.00	0.00	0.00	0.00	0.02	0.59
**24/01/2021**	0.00	0.00	0.00	0.00	0.00	0.00	0.00	0.04	0.80
**31/01/2021**	0.00	0.00	0.00	0.00	0.00	0.00	0.00	0.59	0.88
**07/02/2021**	0.00	0.00	0.00	0.00	0.00	0.00	0.05	0.85	0.95
**14/02/2021**	0.00	0.00	0.00	0.00	0.00	0.00	0.08	0.96	0.95
**21/02/2021**	0.00	0.00	0.00	0.00	0.00	0.00	0.11	0.97	0.94
**28/02/2021**	0.00	0.00	0.00	0.00	0.00	0.00	0.43	0.98	0.95
**07/03/2021**	0.00	0.00	0.00	0.00	0.00	0.00	0.79	0.98	0.95
**14/03/2021**	0.00	0.00	0.00	0.00	0.00	0.27	0.87	0.98	0.95
**21/03/2021**	0.00	0.00	0.00	0.00	0.00	0.73	0.93	0.98	0.95
**28/03/2021**	0.00	0.00	0.00	0.00	0.00	0.87	0.95	0.98	0.95
**04/04/2021**	0.00	0.00	0.00	0.00	0.00	0.90	0.95	0.98	0.95
**11/04/2021**	0.00	0.00	0.00	0.00	0.00	0.91	0.96	0.99	0.95
**18/04/2021**	0.00	0.00	0.00	0.00	0.30	0.92	0.96	0.99	0.95
**25/04/2021**	0.00	0.00	0.00	0.00	0.35	0.92	0.96	0.99	0.95
**02/05/2021**	0.00	0.00	0.00	0.00	0.37	0.92	0.96	0.99	0.95
**09/05/2021**	0.00	0.00	0.00	0.00	0.73	0.93	0.96	0.99	0.95
**16/05/2021**	0.00	0.00	0.00	0.00	0.79	0.93	0.97	0.99	0.95
**23/05/2021**	0.00	0.00	0.00	0.00	0.82	0.94	0.97	0.99	0.95
**30/05/2021**	0.00	0.00	0.00	0.58	0.84	0.94	0.97	0.99	0.95
**06/06/2021**	0.00	0.00	0.00	0.65	0.85	0.94	0.97	0.99	0.94
**13/06/2021**	0.00	0.00	0.20	0.71	0.86	0.95	0.97	0.99	0.94
**20/06/2021**	0.00	0.00	0.53	0.75	0.87	0.95	0.97	0.99	0.94
**27/06/2021**	0.00	0.02	0.54	0.77	0.87	0.95	0.98	0.99	0.94
**04/07/2021**	0.00	0.05	0.61	0.78	0.88	0.95	0.98	0.99	0.94
**11/07/2021**	0.00	0.07	0.64	0.79	0.88	0.95	0.98	0.99	0.94
**18/07/2021**	0.00	0.16	0.67	0.80	0.89	0.95	0.98	0.99	0.94
**25/07/2021**	0.00	0.16	0.68	0.81	0.89	0.96	0.98	0.99	0.94
**Current coverage**	**0.00**	**0.16**	**0.68**	**0.81**	**0.89**	**0.96**	**0.98**	**0.99**	**0.94**

Source: NHS England[16]

Table S10. Forecasted age-dependent vaccination coverage over time (BC scenario)

Date	0-9y	10-19y	20-29y	30-39y	40-49y	50-59y	60-69y	70-79y	80+y
**01/08/2021**	0	0.17	0.70	0.82	0.89	0.96	0.98	0.99	0.94
**08/08/2021**	0	0.17	0.72	0.83	0.90	0.96	0.98	0.99	0.94
**15/08/2021**	0	0.18	0.73	0.84	0.90	0.96	0.98	0.99	0.95
**22/08/2021**	0	0.18	0.75	0.84	0.90	0.96	0.98	0.99	0.95
**29/08/2021**	0	0.19	0.75	0.85	0.90	0.96	0.98	0.99	0.95
**05/09/2021**	0	0.19	0.75	0.85	0.90	0.96	0.98	0.99	0.95
**12/09/2021**	0	0.20	0.75	0.85	0.90	0.96	0.98	0.99	0.95
**19/09/2021**	0	0.20	0.75	0.85	0.90	0.96	0.98	0.99	0.95
**26/09/2021**	0	0.21	0.75	0.85	0.90	0.96	0.98	0.99	0.95
**03/10/2021**	0	0.21	0.75	0.85	0.90	0.96	0.98	0.99	0.95
**10/10/2021**	0	0.22	0.75	0.85	0.90	0.96	0.98	0.99	0.95
**17/10/2021**	0	0.22	0.75	0.85	0.90	0.96	0.98	0.99	0.95
**24/10/2021**	0	0.23	0.75	0.85	0.90	0.96	0.98	0.99	0.95
**31/10/2021**	0	0.23	0.75	0.85	0.90	0.96	0.98	0.99	0.95
**07/11/2021**	0	0.24	0.75	0.85	0.90	0.96	0.98	0.99	0.95
**14/11/2021**	0	0.24	0.75	0.85	0.90	0.96	0.98	0.99	0.95
**21/11/2021**	0	0.24	0.75	0.85	0.90	0.96	0.98	0.99	0.95
**28/11/2021**	0	0.24	0.75	0.85	0.90	0.96	0.98	0.99	0.95
**05/12/2021**	0	0.24	0.75	0.85	0.90	0.96	0.98	0.99	0.95
**12/12/2021**	0	0.24	0.75	0.85	0.90	0.96	0.98	0.99	0.95
**19/12/2021**	0	0.24	0.75	0.85	0.90	0.96	0.98	0.99	0.95
**26/12/2021**	0	0.24	0.75	0.85	0.90	0.96	0.98	0.99	0.95
**Maximum coverage**	0.00	0.24	0.75	0.85	0.90	0.96	0.98	0.99	0.95

Table S11. Forecasted age-dependent vaccination coverage over time (BCA scenario)

Date	0-9y	10-19y	20-29y	30-39y	40-49y	50-59y	60-69y	70-79y	80+y
**01/08/2021**	0	0.19	0.70	0.82	0.89	0.96	0.98	0.99	0.94
**08/08/2021**	0	0.22	0.72	0.83	0.90	0.96	0.98	0.99	0.94
**15/08/2021**	0	0.25	0.73	0.84	0.90	0.96	0.98	0.99	0.95
**22/08/2021**	0	0.28	0.75	0.84	0.90	0.96	0.98	0.99	0.95
**29/08/2021**	0	0.30	0.75	0.85	0.90	0.96	0.98	0.99	0.95
**05/09/2021**	0	0.33	0.75	0.85	0.90	0.96	0.98	0.99	0.95
**12/09/2021**	0	0.36	0.75	0.85	0.90	0.96	0.98	0.99	0.95
**19/09/2021**	0	0.39	0.75	0.85	0.90	0.96	0.98	0.99	0.95
**26/09/2021**	0	0.42	0.75	0.85	0.90	0.96	0.98	0.99	0.95
**03/10/2021**	0	0.44	0.75	0.85	0.90	0.96	0.98	0.99	0.95
**10/10/2021**	0	0.47	0.75	0.85	0.90	0.96	0.98	0.99	0.95
**17/10/2021**	0	0.50	0.75	0.85	0.90	0.96	0.98	0.99	0.95
**24/10/2021**	0	0.53	0.75	0.85	0.90	0.96	0.98	0.99	0.95
**31/10/2021**	0	0.56	0.75	0.85	0.90	0.96	0.98	0.99	0.95
**07/11/2021**	0	0.59	0.75	0.85	0.90	0.96	0.98	0.99	0.95
**14/11/2021**	0	0.59	0.75	0.85	0.90	0.96	0.98	0.99	0.95
**21/11/2021**	0	0.59	0.75	0.85	0.90	0.96	0.98	0.99	0.95
**28/11/2021**	0	0.59	0.75	0.85	0.90	0.96	0.98	0.99	0.95
**05/12/2021**	0	0.59	0.75	0.85	0.90	0.96	0.98	0.99	0.95
**12/12/2021**	0	0.59	0.75	0.85	0.90	0.96	0.98	0.99	0.95
**19/12/2021**	0	0.59	0.75	0.85	0.90	0.96	0.98	0.99	0.95
**26/12/2021**	0	0.59	0.75	0.85	0.90	0.96	0.98	0.99	0.95
**Maximum coverage**	0	0.59	0.75	0.85	0.90	0.96	0.98	0.99	0.95

Table S12. Forecasted age-dependent vaccination coverage over time (BCAC scenario)

Date	0-9y	10-19y	20-29y	30-39y	40-49y	50-59y	60-69y	70-79y	80+y
**01/08/2021**	0.36	0.22	0.70	0.82	0.89	0.96	0.98	0.99	0.94
**08/08/2021**	0.37	0.27	0.72	0.83	0.90	0.96	0.98	0.99	0.94
**15/08/2021**	0.39	0.33	0.73	0.84	0.90	0.96	0.98	0.99	0.95
**22/08/2021**	0.40	0.38	0.75	0.84	0.90	0.96	0.98	0.99	0.95
**29/08/2021**	0.41	0.44	0.75	0.85	0.90	0.96	0.98	0.99	0.95
**05/09/2021**	0.42	0.49	0.75	0.85	0.90	0.96	0.98	0.99	0.95
**12/09/2021**	0.43	0.55	0.75	0.85	0.90	0.96	0.98	0.99	0.95
**19/09/2021**	0.44	0.60	0.75	0.85	0.90	0.96	0.98	0.99	0.95
**26/09/2021**	0.46	0.66	0.75	0.85	0.90	0.96	0.98	0.99	0.95
**03/10/2021**	0.47	0.71	0.75	0.85	0.90	0.96	0.98	0.99	0.95
**10/10/2021**	0.48	0.77	0.75	0.85	0.90	0.96	0.98	0.99	0.95
**17/10/2021**	0.49	0.82	0.75	0.85	0.90	0.96	0.98	0.99	0.95
**24/10/2021**	0.50	0.88	0.75	0.85	0.90	0.96	0.98	0.99	0.95
**31/10/2021**	0.51	0.93	0.75	0.85	0.90	0.96	0.98	0.99	0.95
**07/11/2021**	0.52	0.99	0.75	0.85	0.90	0.96	0.98	0.99	0.95
**14/11/2021**	0.52	1.00	0.75	0.85	0.90	0.96	0.98	0.99	0.95
**21/11/2021**	0.52	1.00	0.75	0.85	0.90	0.96	0.98	0.99	0.95
**28/11/2021**	0.52	1.00	0.75	0.85	0.90	0.96	0.98	0.99	0.95
**05/12/2021**	0.52	1.00	0.75	0.85	0.90	0.96	0.98	0.99	0.95
**12/12/2021**	0.52	1.00	0.75	0.85	0.90	0.96	0.98	0.99	0.95
**19/12/2021**	0.52	1.00	0.75	0.85	0.90	0.96	0.98	0.99	0.95
**26/12/2021**	0.52	1.00	0.75	0.85	0.90	0.96	0.98	0.99	0.95
**Maximum coverage**	**0.52**	**1.00**	**0.75**	**0.85**	**0.90**	**0.96**	**0.98**	**0.99**	**0.95**

FIGURES

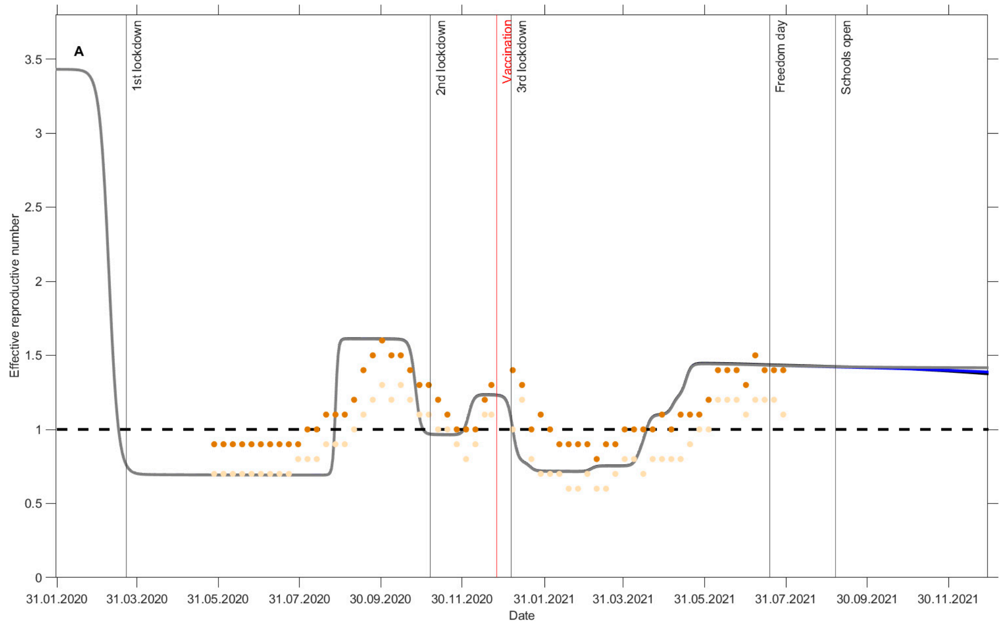

Figure S1: The effective reproductive number over time since the beginning of the pandemic and assumed level for model predictions. Dots represent the observed lower and upper values of the effective reproductive number in the UK

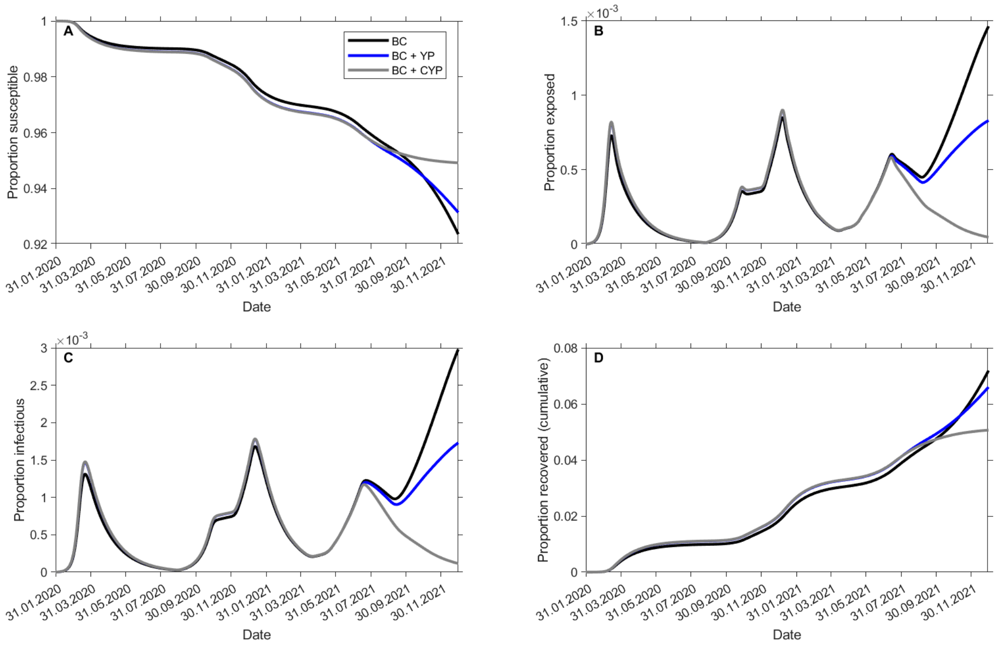

Figure S2: Model trace showing the proportion of individuals who were susceptible, exposed, infectious and those who recovered from SARS-CoV-2 infection in the UK population up to December 2021.

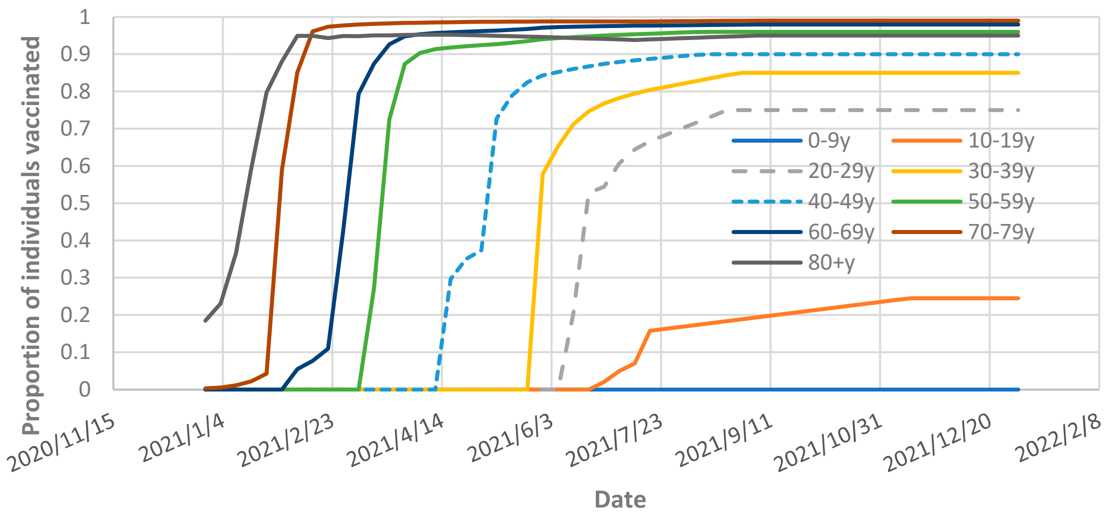

Figure S3: Age dependent vaccine coverage in adults ≥18 years of age and vulnerable adolescents (BC).

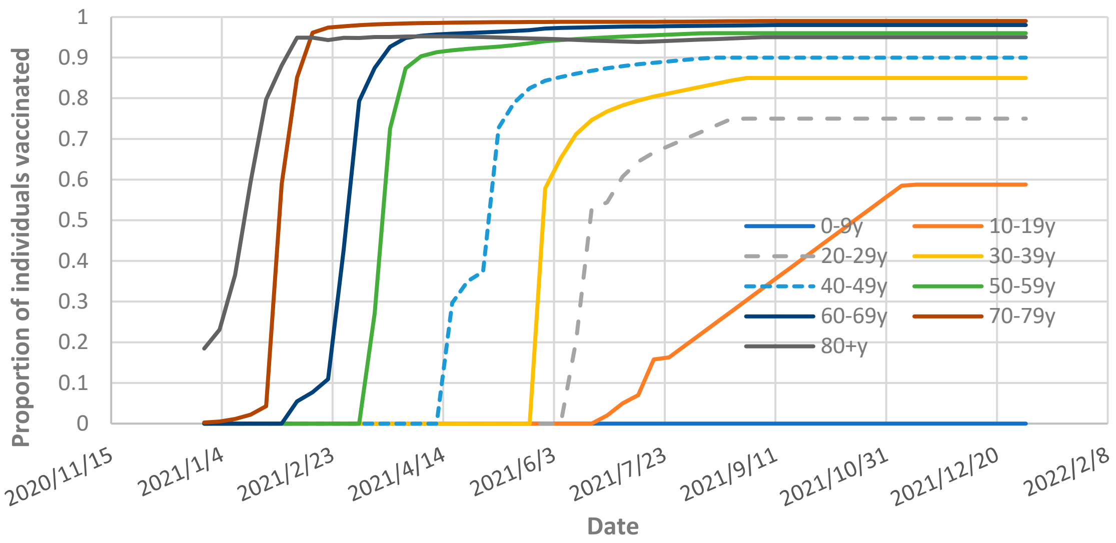

Figure S4: Age dependent vaccine coverage in adults and all adolescents (BCA).

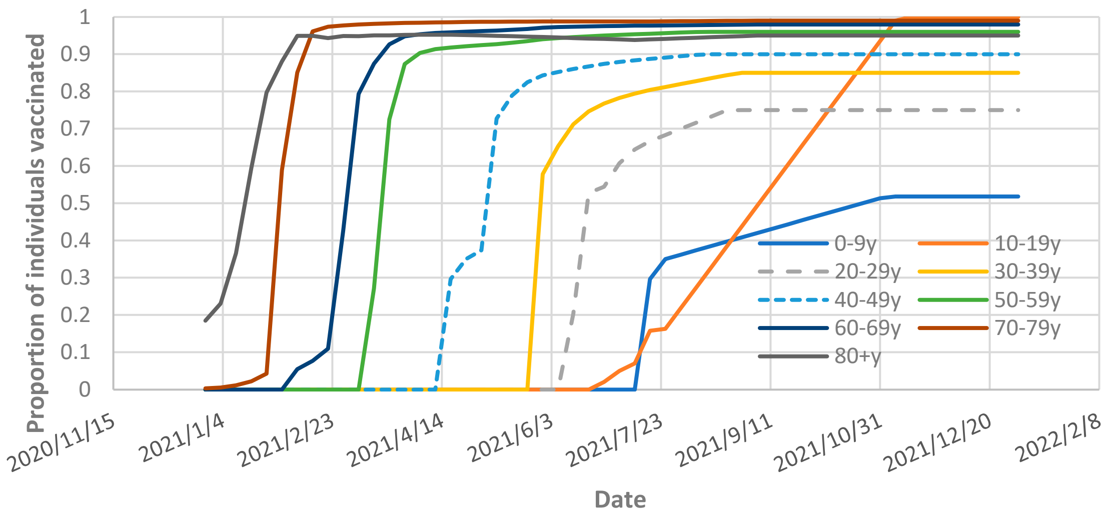

Figure S5: Age dependent vaccine coverage in adults, adolescents and children (BCAC).

REFERENCES

1. Salje H, Tran Kiem C, Lefrancq N, Courtejoie N, Bosetti P, Paireau J, et al. Estimating the burden of SARS-CoV-2 in France. Science. 2020;369(6500):208-11.
2. Diekmann O, Heesterbeek JA, Metz JA. On the definition and the computation of the basic reproduction ratio R0 in models for infectious diseases in heterogeneous populations. J Math Biol. 1990;28(4):365-82.
3. Wölfel R, Corman VM, Guggemos W, Seilmaier M, Zange S, Müller MA, et al. Virological assessment of hospitalized patients with COVID-2019. Nature. 2020;581(7809):465-9.
4. Imai N, Cori A, Dorigatti I, Baguelin M, Donnelly CA, Riley S, et al. Transmissibility of 2019-nCoV. Imperial College London (25-01-2020). 2021. Available from: https://doi.org/10.25561/77148.
5. Bernal JL, Andrews N, Gower C, Gallagher E, Simmons R, Thelwall S, et al. Effectiveness of COVID-19 vaccines against the B.1.617.2 variant. medRxiv. 2021:2021.05.22.21257658.
6. GOV.UK. Press release: One dose of COVID-19 vaccine can cut household transmission by up to half. 2021. Available from: https://www.gov.uk/government/news/one-dose-of-covid-19-vaccine-can-cut-household-transmission-by-up-to-half.
7. Office for National Statistics. Prevalence of ongoing symptoms following coronavirus (COVID-19) infection in the UK: 1 July 2021. 2021. Available from: https://www.ons.gov.uk/peoplepopulationandcommunity/healthandsocialcare/conditionsanddiseases/bulletins/prevalenceofongoingsymptomsfollowingcoronaviruscovid19infectionintheuk/1july2021.
8. Office for National Statistics. Population estimates by output areas, electoral, health and other geographies, England and Wales: mid-2019. 2020 19 July 2021. Available from: https://www.ons.gov.uk/peoplepopulationandcommunity/populationandmigration/populationestimates/bulletins/annualsmallareapopulationestimates/mid2019.
9. Mossong J, Hens N, Jit M, Beutels P, Auranen K, Mikolajczyk R, et al. Social Contacts and Mixing Patterns Relevant to the Spread of Infectious Diseases. PLOS Medicine. 2008;5(3):e74.
10. Department of Health and Social Care. The R value and growth rate. Department of Health and Social Care. 2021.
11. Ferguson N, Laydon D, Nedjati Gilani G, Imai N, Ainslie K, Baguelin M, et al. Report 9: Impact of non-pharmaceutical interventions (NPIs) to reduce COVID19 mortality and healthcare demand. 2020. Available from: https://www.imperial.ac.uk/media/imperial-college/medicine/sph/ide/gida-fellowships/Imperial-College-COVID19-NPI-modelling-16-03-2020.pdf.
12. Smith C, Odd D, Harwood R, Ward J, Linney M, Clark M, et al. Deaths in Children and Young People in England following SARS-CoV-2 infection during the first pandemic year: a national study using linked mandatory child death reporting data. medRxiv. 2021:2021.07.07.21259779.
13. Office for National Statistics. Coronavirus and the social impacts of 'long COVID' on people's lives in Great Britain. 2021. Available from: https://www.ons.gov.uk/peoplepopulationandcommunity/healthandsocialcare/conditionsanddiseases/articles/coronavirusandthesocialimpactsoflongcovidonpeopleslivesingreatbritain/7aprilto13june2021.
14. Wolfel R, Corman VM, Guggemos W, Seilmaier M, Zange S, Muller MA, et al. Virological assessment of hospitalized patients with COVID-2019. Nature. 2020;581(7809):465-9.
15. Public Health England. COVID-19 vaccine surveillance report - Week 28. 2021 19 July 2021. Available from: https://assets.publishing.service.gov.uk/government/uploads/system/uploads/attachment_data/file/1002580/Vaccine_surveillance_report_-_week_28.pdf.
16. NHS England. COVID-19 Vaccinations. 2021. Available from: https://www.england.nhs.uk/statistics/statistical-work-areas/covid-19-vaccinations/.
